# Synthesis, Characterization and Reactions of (Azidoethynyl)trimethylsilane

**DOI:** 10.3390/molecules201219770

**Published:** 2015-12-01

**Authors:** Klaus Banert, Manfred Hagedorn, Zhuang Wu, Xiaoqing Zeng

**Affiliations:** 1 Organic Chemistry, Chemnitz University of Technology, Strasse der Nationen 62, Chemnitz 09111, Germany; manfred.hagedorn@chemie.tu-chemnitz.de; 2 College of Chemistry, Chemical Engineering and Materials Science, Soochow University, 199 Ren-Ai Road, Suzhou Industrial Park, Suzhou 215123, China; 20144209141@stu.suda.edu.cn

**Keywords:** carbenes, ethynyl azides, gas phase IR data, photolysis, reactive intermediates

## Abstract

Synthesis of azido(trimethylsilyl)acetylene (**6**) was performed by treating the iodonium salt **5** with highly soluble hexadecyltributylphosphonium azide (QN_3_) at −40 °C. Although this product is very unstable, it can nevertheless be trapped by the click reaction with cyclooctyne to give the corresponding 1,2,3-triazole, and also directly characterized by ^1^H- and ^13^C-NMR data as well as IR-spectra, which were measured in solution at low temperature and in the gas phase. The thermal or photochemical decay of azide **6** leads to cyano(trimethylsilyl)carbene. This is demonstrated not only by quantum chemical calculations, but also by the trapping reactions with the help of isobutene.

## 1. Introduction

**Scheme 1:**
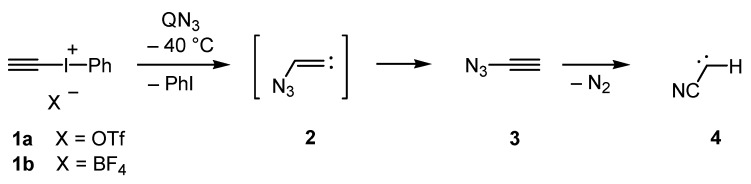
Synthesis and decay of azidoacetylene (**3**).

Whereas vinyl [1,2,3,4,5,6,7] and allenyl azides [7,8] are well-known for their manifold reactions and can be prepared by various methods, all attempts to isolate or even to simply detect ethynyl azides were unsuccessful until quite recently. The experimental pursuit of 1-azidoalk-1-ynes dates back to 1910 with the work of Forster and Newman [9]; however, like all other early attempts, only unwanted products were obtained, and no conclusive evidence for the formation of ethynyl azides or their secondary decay reactions was found [7,10]. This class of azides thus remained elusive for more than 100 years. Recently, trapping of phenylethynyl azide by 1,3-dipolar cycloaddition has been reported after *in situ* generation of this azide in the presence of highly reactive cyclooctyne. It was additionally shown that 1-azidoalk-1-ynes are short-lived species, which tend to cleave off dinitrogen with formation of the corresponding cyanocarbene [11]. Finally, the parent azidoacetylene (**3**) was synthesized by treatment of iodonium salts **1a** or **1b** with a highly soluble azide source, such as hexadecyltributylphosphonium azide (QN_3_) [12], at −40 °C (Scheme 1) [13]. Iodonium salts similar to **1a**,**b** are well-known to react with azide at the terminal acetylenic carbon atom to generate azidovinylidene intermediates after liberation of iodobenzene [14].Thus, it is plausible that exposure of **1a**,**b** to QN_3_ first leads to azidovinylidene (**2**), which undergoes a Fritsch-Buttenberg-Wiechell-like rearrangement to produce **3** [13]. In solution at low temperature, the unique azide **3** was characterized by NMR and IR spectroscopy. The latter method was also utilized to analyze unstable **3** in the gas phase or in argon matrix, and to monitor the thermal or photochemical decay to generate cyanocarbene **4** [15,16]. In the case of the parent compound **3**, previous *ab initio* studies predicted the highest barrier for the loss of dinitrogen, whereas ethynyl azides with a substituent at C-2 should more easily undergo the cleavage reaction [17]. Especially, 1-azidoethynes with a donor substituent at C-2, such as amino or ethylsulfanyl, are calculated to be extremely unstable; however, a silyl group in this position should lead to an azide with only slightly reduced stability if compared with that of **3**.

Unfortunately, the parent compound **3** is a very explosive substance, and even recondensation of its solution under reduced pressure can lead to an explosion. Here, we report on trimethylsilylethynyl azide (**6**), which is significantly less explosive and did not cause any incident during the experiments.

## 2. Results and Discussion

**Scheme 2:**
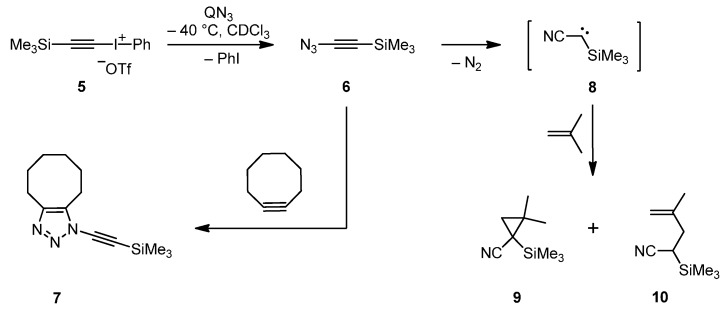
Synthesis, trapping and decay of (azidoethynyl)trimethylsilane (**6**).

When we monitored the reaction of the known [18] iodonium salt **5** with QN_3_ in deuterated chloroform at −40 °C by ^1^H-NMR spectroscopy, nearly quantitative formation of the azide **6** was observed (Scheme 2). Thus, it was not surprising that the trapping product **7** was isolated in high yield (89%) after treating the reaction mixture with cyclooctyne. Recondensation of the reaction mixture with **6** led a solution of this azide including a part of the byproduct iodobenzene but without any salts or other reagents; however, ^1^H-NMR analyses showed a reduced yield of only 10%–54%, even when the recondensation was performed at −40 °C with the help of an oil diffusion pump (10^−6^ Torr). There may be two reasons for this loss of **6**: since **6** is obviously less volatile than **3**, the thermal stress in the recondensation process should be higher, and the intrinsic stability of **6** is possibly lower than that of **3**. In order to clarify this, we determined the half-life periods of **6** in deuterated chloroform at −20 °C (35 min) and −30 °C (230 min) by ^1^H-NMR spectroscopy. These results indicate a lower thermal stability of **6** compared to that of the parent compound **3**, for which a half-life period of approximately 17 hours in dichloromethane at −30 °C was reported [13]. Nevertheless, **7** was formed in 95% yield (^1^H-NMR), when a recondensed solution of **6** was treated with an excess of cyclooctyne.

The title compound **6** was characterized not only by ^1^H-NMR spectroscopy and the trapping reaction with cyclooctyne, but also by ^13^C-NMR data and IR spectra measured at low temperature in solution. In agreement with the corresponding chemical shifts of acetylene [19], trimethylsilylacetylene [20] and **3** [13], the ^13^C-NMR signals of **6** at δ = 68.3 and 89.9 (ppm) were assigned to the C–Si and C–N_3_ sp-hybridized carbons, respectively. This assignment was also supported by ^13^C,^1^H long-range shift correlation experiments (GHMBC AD and CIGAR, see the Supplementary Materials), and the upfield shift for the signal of the C–Si carbon indicates that the azido group acts as a π donor, through which **6** becomes an electron-rich alkyne. When the decay of **6** in deuterated chloroform was monitored by IR spectroscopy in the temperature range of −60 to +20 °C, three characteristic signals at 2173 (weak, C≡C), 2103 (very strong, N_3_/asym) and 1254 cm^−1^ (medium, N_3_/sym) were detected. By increasing the temperature, these signals became weaker, and at −10 °C the decay of the azide **6** was nearly complete. The IR data of **6** are similar to the corresponding frequencies which were observed for **3** after measurement in chloroform at −20 °C [13]. In the latter case, however, the intensity pattern was quite different owing to strong vibrational coupling by Fermi resonances [15].

**Figure 1:**
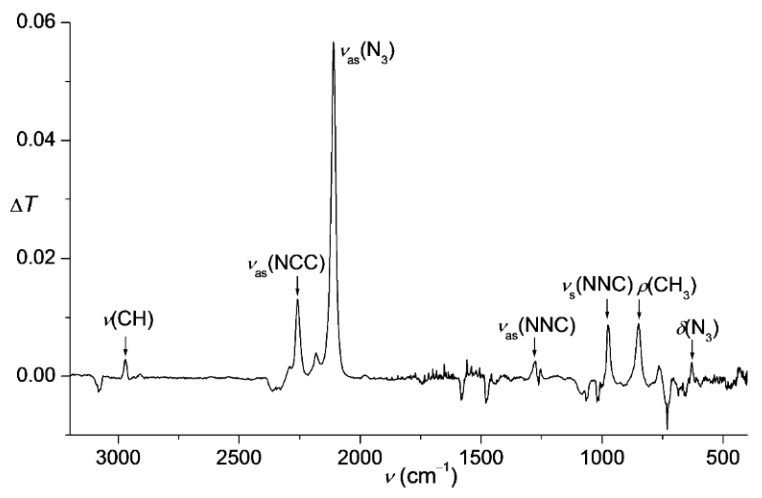
IR difference spectrum showing the changes before and after thermal decomposition of gaseous Me_3_SiCCN_3_ (**6**) in the range of 3200–400 cm^−1^. The bands pointing downwards belong to unidentified decomposition product. Band positions of **6** are 2970, 2259, 2183, 2110, 1277, 976, 850, 629 cm^−1^.

**Figure 2:**
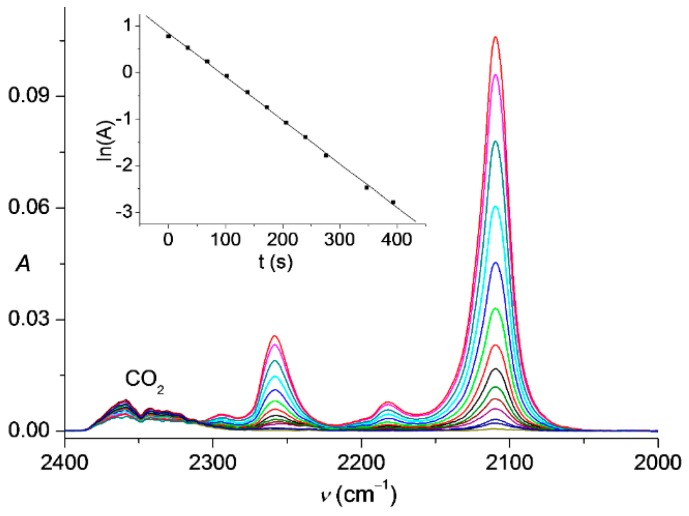
IR spectra of gaseous Me_3_SiCCN_3_ (**6**) in the range of 2400–2000 cm^−1^ showing its thermal decay at 282 K in 680 s. Inset: First-order kinetics for the decomposition, the corresponding rate constant is (9.36 ± 0.11) × 10^−3^ s^−1^ and half-life time is 74 s.

When the title compound **6** was generated from **5** and QN_3_ in the high-boiling solvent propylene carbonate instead of chloroform, volatile **6** could be slowly distilled into a cold glass trap (–65 °C) and thereafter quickly vaporized into an IR gas cell to measure the corresponding spectra (Figure 1). The decay of gaseous **6** was also monitored by IR spectroscopy which indicated a significantly more rapid decomposition of this azide if compared to the analogous experiments with **3** [16] (Figure 2).

**Figure 3:**
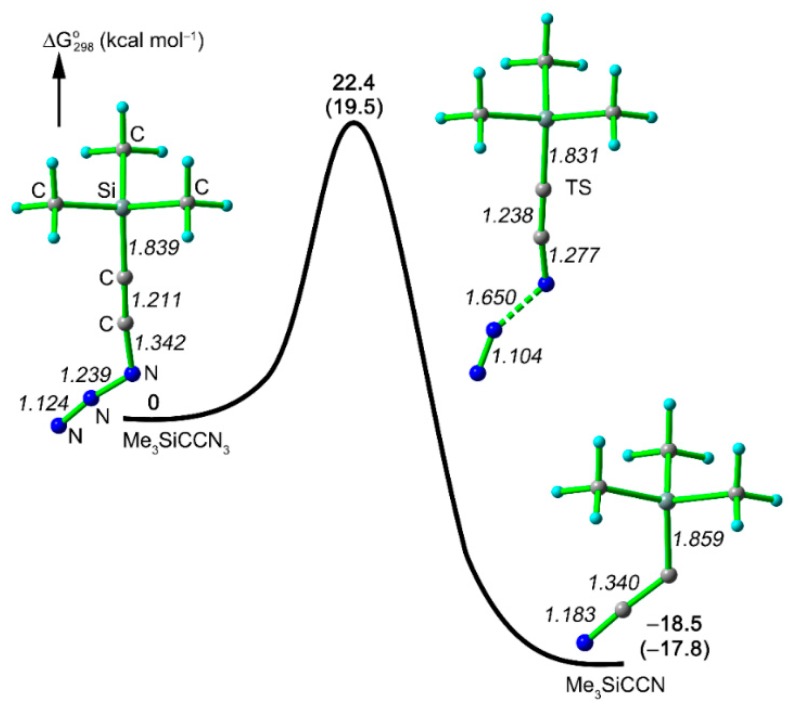
Calculated potential energy surface for the decomposition of Me_3_SiCCN_3_ (**6**) to generate carbene **8** at the CBS-QB3 and B3LYP/6-311++G(3df,3pd) (in parentheses) levels of theory. The selected bond lengths (Å, italics) calculated at the B3LYP/6-311++G(3df,3pd) level are also shown.

The decomposition of ethynyl azide **6** is expected to lead to cyano(trimethylsilyl)carbene (**8**). The formation of this short-lived species is also supported by quantum chemical calculations that explained the generation of **8** from **6** through a synchronous reaction without a nitrene intermediate [17,21] (Figure 3). The activation barrier for this process was calculated to amount 20–22 kcal·mol^−1^, and such a low value corresponds to the fact that **6** is a highly unstable compound.

Since the decay of the title compound **6** was postulated to lead to carbene **8**, we tried to trap the latter very reactive intermediate by storing a recondensed solution of **6** for 16 h at low temperature (−40 to + 20 °C) in the presence of a great excess of isobutene. After work-up, we isolated a mixture of the cyclopropanation product **9** (69% based on **6**) and the insertion product **10** (5%); and similar yields (56% and 5% based on **5**) were achieved when the reaction mixture resulting from treatment of **5** with QN_3_ was directly diluted with a large amount of isobutene (Scheme 2). Irradiation of a recondensed solution of **6** in the presence of an excess of isobutene at −60 °C led also to **9** and **10**; however, the yields were lower. The cyclopropane derivative **9** is a new compound, whereas **10** was previously prepared by an alternative method [22].

## 3. Experimental Section

*Caution!* Small covalent azides are potentially hazardous and explosive. Me_3_SiCCN_3_ (**6**) was found to be highly unstable, and the neat substance should only be handled on milligram scales, and appropriate safety precautions (face shields, leather gloves, and protective leather clothing) are strongly recommended. For potential hazards in handling hydrazoic acid and organic azides, see [23].

### 3.1. General Information

#### 3.1.1. Apparatus

FTIR spectra were recorded with a Nicolet iS5 spectrophotometer (Thermo Scientific Inc., Waltham, MA, USA) and solutions in KBr cuvettes. Low-temperature FTIR spectra with **6** in solution were recorded with a Nicolet 6700 spectrophotometer (Thermo Scientific). ^1^H-NMR spectra were recorded with a Unity Inova 400 spectrometer (Varian Inc., Palo Alto, CA, USA) operating at 400 MHz. By using the same spectrometer, ^13^C-NMR data were recorded at 100.6 MHz. NMR signals were referenced to TMS (δ = 0) or solvent signals and recalculated relative to TMS. The multiplicities of ^13^C-NMR signals were determined with the aid of DEPT135 experiments. HRMS (ESI) spectra were recorded with a micrOTOF-QII spectrometer (Bruker-Daltronik GmbH, Bremen, Germany). TLC was performed with Polygram SIL G/UV_254_ polyester sheets (Macherey-Nagel, Düren, Germany). Photolysis experiments were executed by using a high-pressure mercury lamp (TQ 150, Heraeus, Hanau, Germany). Reactions in connection with IR analysis of gaseous **6** were performed in a two-bulb glass vessel (volume 2 × 30 mL), which was connected to the vacuum line and equipped with small magnetic stir bars and a J-Young PTFE stem. Volatile materials were manipulated in a glass vacuum line equipped with a pressure gauge (CTR100, Oerlikon Leybold, Köln, Germany) and three U-traps. The vacuum line was connected to an IR gas cell (optical path length 20 cm, Si windows, 0.3 mm thick), fitted into the sample compartment of the FT-IR instrument (Tensor 27, Bruker, Billerica, MA, USA) for measuring gas-phase IR spectra at a resolution of 2 cm^−1^.

#### 3.1.2. Chemicals

Trimethylsilylethynyl(phenyl)iodonium triflate (**5**) [18], hexadecyltributylphosphonium azide (QN_3_) [12] and cyclooctyne [24] were prepared according to the literature. Propylene carbonate (99.5%, Acros, Morris Plains, NJ, USA) was degassed before use.

### 3.2. Synthesis of (Azidoethynyl)trimethylsilane *(**6**)* in Solution

A solution of QN_3_ (611 mg, 1.3 mmol) in CDCl_3_ (3 mL) and CH_2_Cl_2_ (20 μL, internal standard) was cooled to −40 °C. Compound **5** (422 mg, 1.0 mmol) was added in portions, and the mixture was stirred at −40 °C for 1 h. During this time, the color of the solution changed to yellow. With the aid of an oil diffusion pump (10^−6^ Torr), volatile **6** and the solvent were recondensed into another flask cooled by liquid nitrogen. This led to a solution of **6** (yield *ca.* 10%–55%, ^1^H-NMR), which additionally contained a part of the iodobenzene. *Caution!* During recondensation, explosion of **6** is possible, so protective shield use is highly recommended. After recondensation, solutions of **6** in CDCl_3_ were utilized for NMR spectroscopy at low temperature. Alternatively, they were diluted with isobutene (50 mL) and slowly warmed to room temperature or irradiated with a high-pressure mercury lamp at −60 °C for 30 min to generate the trapping products **9** and **10**. Furthermore, solutions of **6** in CDCl_3_ were treated at −40 °C with a solution of an excess of cyclooctyne in the same solvent to produce the triazole **7**. ^1^H-NMR (CDCl_3_) δ = 0.13 (s, 9H, Si*Me_3_*). ^13^C-NMR (CDCl_3_) δ = −0.18 (q, Si*Me_3_*), 68.67 (s, Me_3_Si-*C*≡C), 90.00 (s, C≡*C*-N_3_). Signal assignment was supported by gHMBC AD and CIGAR experiments. IR (CDCl_3_, −60 °C) $\tilde{\nu }$ = 2173 (w), 2103 (s), 1254 (m) cm^−1^.

### 3.3. Synthesis of Gaseous (Azidoethynyl)trimethylsilane *(**6**)*

Trimethylsilylethynyl(phenyl)iodonium triflate (**5**) (149 mg, 0.33 mmol) and hexadecyltributyl-phosphonium azide (QN_3_, 340 mg, 0.7 mmol) were placed into the two separate bulbs of the reaction vessel. The reaction vessel was then connected to the vacuum line, and propylene carbonate (3 mL) was added to each bulb under argon gas protection. The reaction vessel was then carefully evacuated and cooled in an ethanol bath (–30 °C). Afterwards, the cold solution of iodonium salt was slowly poured into the other solution in portions, while the vessel was always under vacuum and volatile products were distilled into three cold traps which were kept at −20, −65, and −196 °C, respectively. The formation of Me_3_SiCCN_3_ (**6**) was evidenced by the occurrence of a white solid in the second trap, and non-condensable N_2_ gas was immediately produced upon mixing. One hour later, in the second trap small amounts of white solid were retained and directly evaporated into the gas cell for IR spectroscopy measurement.

### 3.4. Synthesis of 1-(Trimethylsilylethynyl)-4,5,6,7,8,9-hexahydro-1H-cycloocta[d][1,2,3]triazole *(**7**)*

A solution of QN_3_ (1035 mg, 2.3 mmol) in CDCl_3_ (8 mL) was cooled to −40 °C. Compound **5** (844 mg, 2 mmol) was added in portions, and the mixture was stirred for 60 min. Thereafter, a solution of cyclooctyne (440 mg, 4 mmol) in CDCl_3_ (1 mL) was added, and the mixture was stirred at −40 °C for 7 h and stored at −30 °C overnight. After dilution with Et_2_O, the reaction mixture was filtered through silica gel to remove the salts. Then the solvents and the excess of cyclooctyne were removed under vacuum, followed by flash chromatography (SiO_2_/Et_2_O) to get **7** (330 mg, 89%) as a yellow oil. ^1^H-NMR (CDCl_3_): δ = 0.25 (s, 9H, Si*Me_3_*), 1.40–1.52 (m, 4H, H-6, H-7), 1.73 (*pseudo* quint, *J* = 6.2 Hz, 2H, H-5), 1.82 (*pseudo* quint, *J* = 6.2 Hz, 2H, H-8), 2.79–2.91 (m, 4H, H-4, H-9). ^13^C-NMR (CDCl_3_): δ = −0.42 (q, Si*Me_3_*), 21.92 (t, C-9), 24.15 (t, C-4), 24.59 (t, CH_2_), 25.44 (t, C-8), 25.85 (t, CH_2_), 27.49 (t, C-5), 82.89 (s, C≡C), 87.66 (s, C≡C), 137.41 (s, C-9a), 142.41 (s, C-3a). IR (CCl_4_): $\tilde{\nu }$ = 2935, 2856, 2190 (C≡C), 1253 cm^−1^. HRMS: *m*/*z* calcd. for C_13_H_21_N_3_Si ([M + H]^+^): 248.1583, found: 248.1564; calcd. for ([M + Na]^+^): 270.1402, found: 270.1382.

### 3.5. Synthesis of 2,2-Dimethyl-1-trimethylsilyl-cyclopropanecarbonitrile *(**9**)* and 4-Methyl-2-trimethylsilyl-pent-4-enenitrile *(**10**)*

A solution of QN_3_ (1035 mg, 2.3 mmol) in CDCl_3_ (8 mL) was cooled to −40 °C. Compound **5** (844 mg, 2 mmol) was added in portions, and the mixture was stirred for 60 min. Thereafter, this solution was added in one portion to recondensed isobutene (100 mL), and the mixture was slowly warmed to room temperature overnight. After dilution with Et_2_O, the reaction mixture was filtered through silica gel to remove the salts. The oily residue was used for flash chromatography (silica gel, Et_2_O/hexane 1:9) to give a pale yellow oil (220 mg, 61%) of a mixture of the nitriles **9** and **10** (10:1). The spectroscopic data of **10** were identical with those already published [22].

**9**: ^1^H-NMR (CDCl_3_): δ = 0.18 (s, 9H, Si*Me_3_*), 0.94 (d, ^2^*J* = 4.2 Hz, 1H, H-3), 1.11 (d ^2^*J* = 4.2 Hz, 1H, H-3), 1.17 (s, 3H, Me), 1.38 (s, 3H, Me). ^13^C-NMR (CDCl_3_): δ = −0.95 (q, Si*Me*_3_), 8.08 (s, C-1), 22.51 (q, Me), 25.20 (q, Me), 25.47 (s, C-2), 26.84 (t, C-3), 112.48 (s, CN), HRMS: *m*/*z* calcd. for C_9_H_17_NSi ([M + Na]^+^): 270.1402, found: 270.1382.

**10**: ^1^H-NMR (CDCl_3_): δ = 0.18 (s, 9H, Si*Me_3_*), 1.74 (br. s, Me), 1.91 (dd, ^3^*J* = 11.7 Hz, ^3^*J* = 4.7 Hz, 1H, C*H*CN), 2.09−2.25 (m, 2H, CH_2_), 4.83–4.87 (m, 2H, =CH_2_). ^13^C-NMR (CDCl_3_): δ = −3.30 (q, Si*Me_3_*), 17.41 (d, C-2), 21.72 (q, CH_3_), 34.55 (t, C-3), 112.48 (s, CN), 123.83 (t, C-5), 142.40 (s, C-4).

### 3.6. Quantum Chemical Calculations

Geometry optimizations and harmonic frequency calculations were performed using the B3LYP [25] method with the 6-311++G(3df,3pd) basis set. Complete basis method (CBS-QB3) was also used [26]. Temperature corrections were all made at 298.15 K. Local minima were confirmed by harmonic vibrational frequencies. The transition states were characterized by a single imaginary frequency. All the calculations were performed using the Gaussian 09 software package [27].

## 4. Conclusions

In summary, we have demonstrated that (azidoethynyl)trimethylsilane (**6**) belongs among the very rare alk-1-ynyl azides which can be detected and spectroscopically characterized. The synthesis of this short-lived compound is easily performed if appropriate substrate, reagent and reaction conditions are used. Because *in situ* generation of azidoacetylenes has recently been utilized to prepare a variety of products via the decomposition to the corresponding cyanocarbenes [11,13,28,29,30,31], the decay of **6** or similar silylethynyl azides to give cyano(silyl)carbenes may be useful in organic synthesis, especially since the latter carbenes are, to the best of our knowledge, unknown in literature.

## Supplementary Materials

Supplementary materials can be accessed at: http://www.mdpi.com/1420-3049/20/12/19770/s1.
