# Supplementary material for: Synthesis, Characterization and Reactions of (Azidoethynyl)trimethylsilane
Source: Molecules. 2015 Dec 1;20(12):21328–35. doi: 10.3390/molecules201219770 (PMC6332308; doi:10.3390/molecules201219770)
Supplement: Supplementary file 1 [file molecules-20-19770-s001.pdf]

Supplementary Materials: Synthesis, Characterization and Reactions of (Azidoethynyl)trimethylsilane

Klaus Banert, Manfred Hagedorn, Zhuang Wu and Xiaoqing Zeng

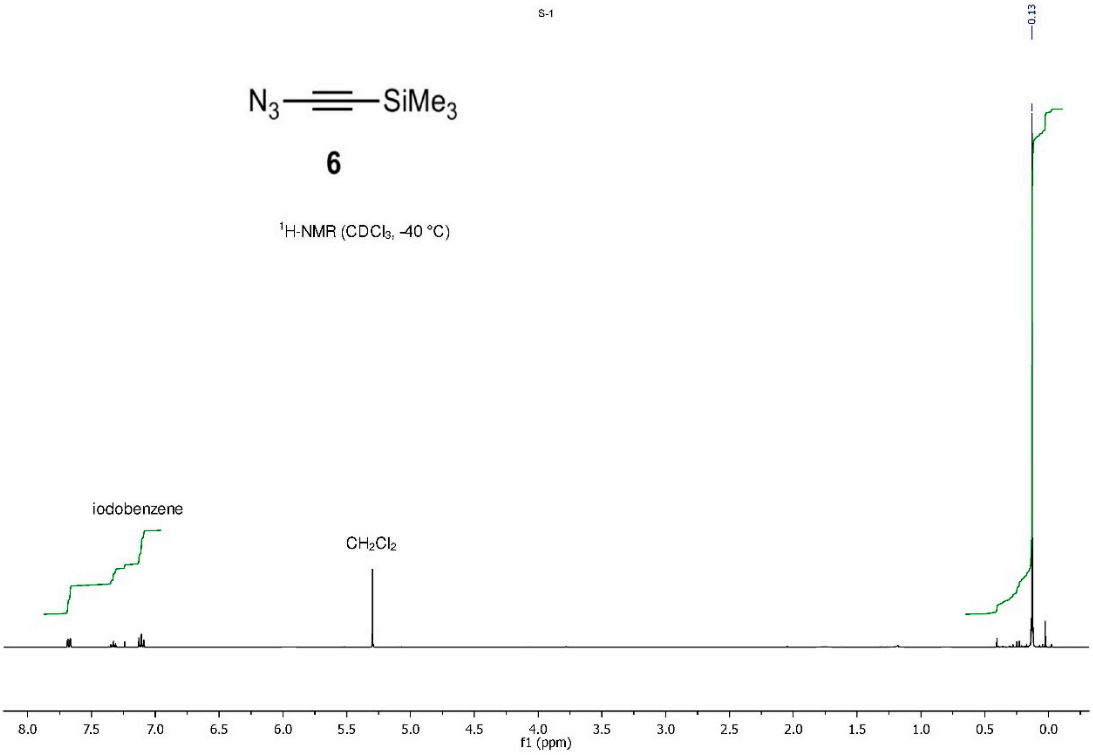

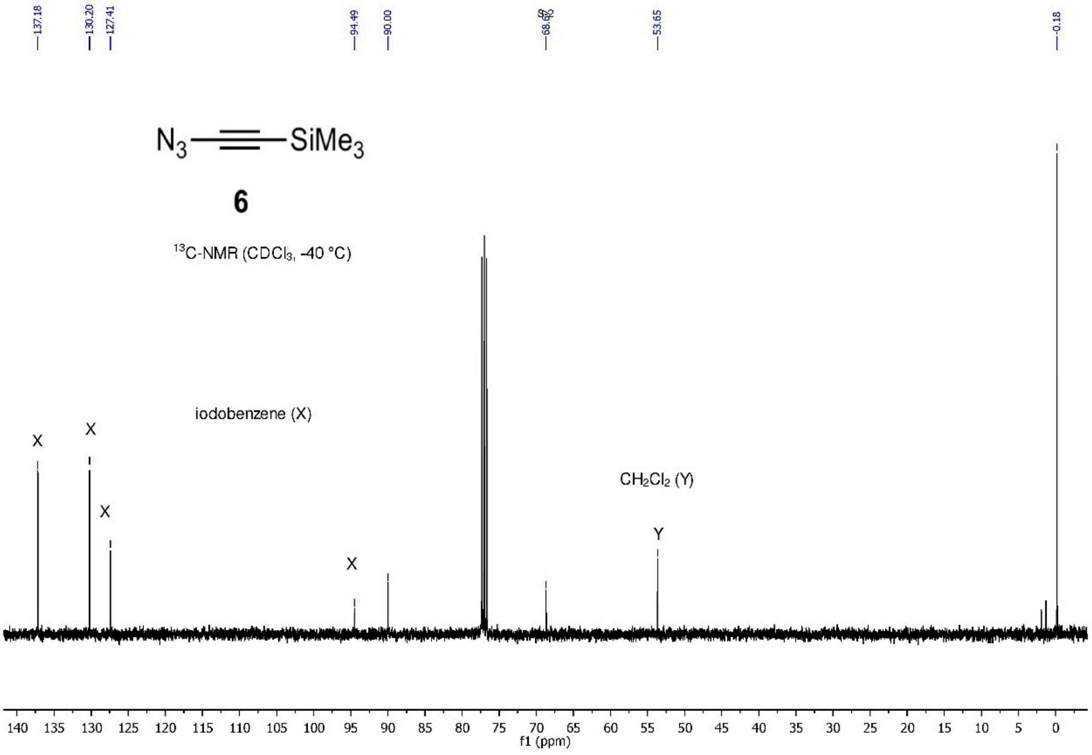

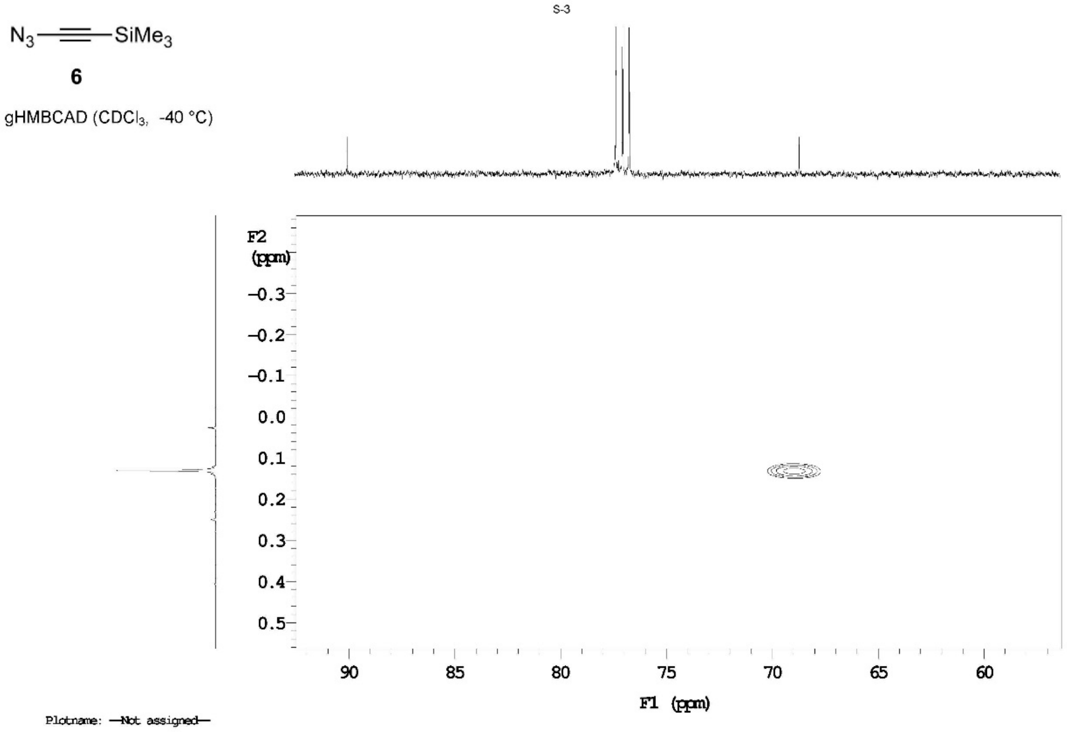
S-3
N3—≡—SiMe3
6
gHMBCAD (CDCl3, -40 °C)
F2 (ppm)
-0.3
-0.2
-0.1
0.0
0.1
0.2
0.3
0.4
0.5
90
85
80
75
70
65
60
F1 (ppm)
Plotname: —Not assigned—

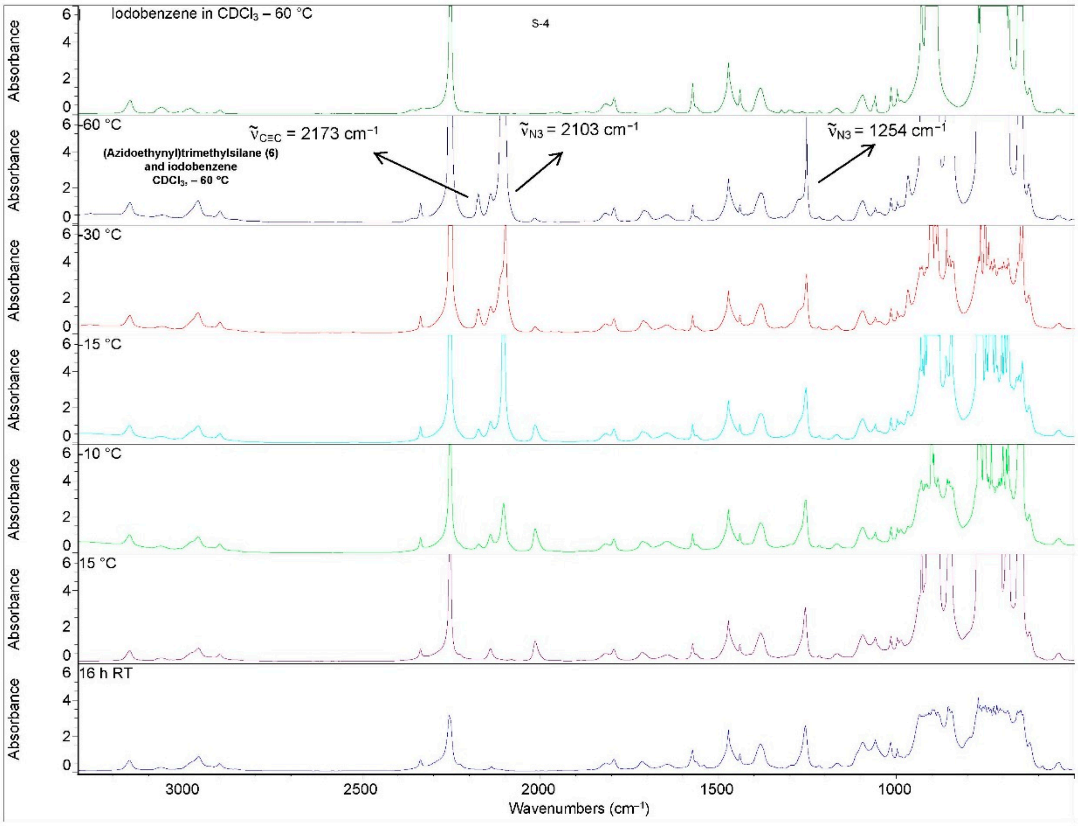
S-4
Iodobenzene in CDCl3 – 60 °C
-60 °C
(Azidoethynyl)trimethylsilane (6) and iodobenzene CDCl3, – 60 °C
ṽC≡C = 2173 cm-1
ṽN3 = 2103 cm-1
ṽN3 = 1254 cm-1
-30 °C
-15 °C
-10 °C
15 °C
16 h RT
Absorbance
Wavenumbers (cm-1)
3000
2500
2000
1500
1000

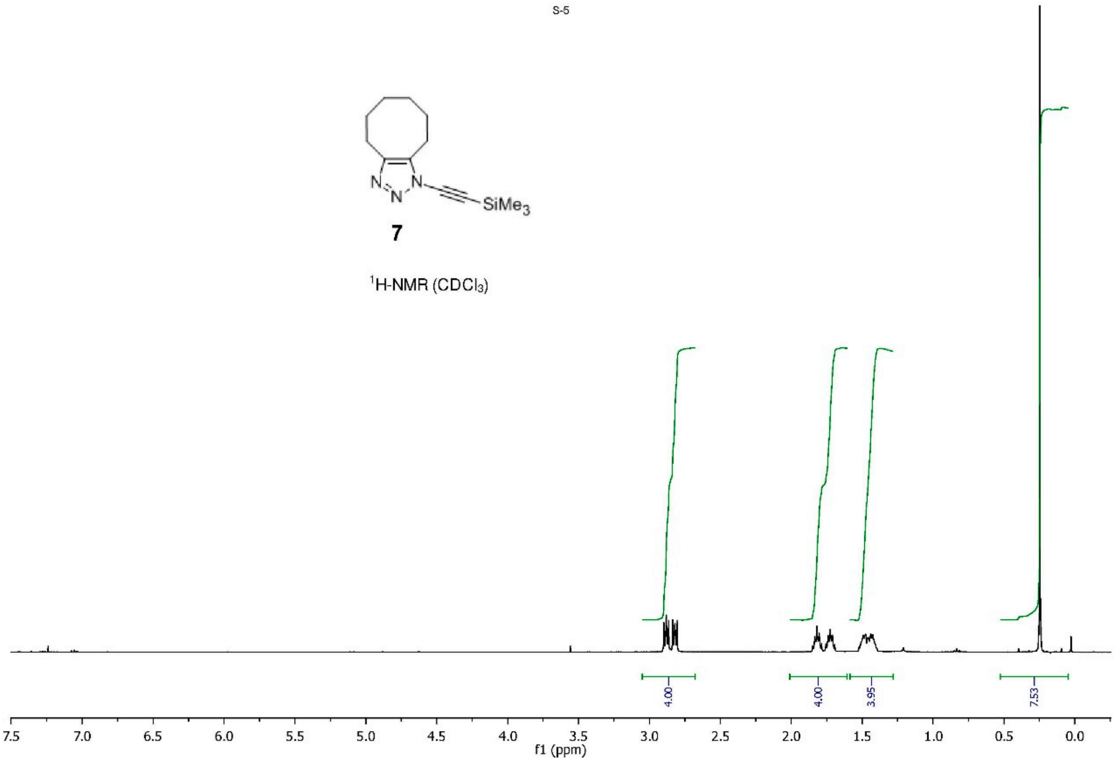
S-5
7
1H-NMR (CDCl3)
4.00
4.00
3.95
7.53
7.5 7.0 6.5 6.0 5.5 5.0 4.5 4.0 3.5 3.0 2.5 2.0 1.5 1.0 0.5 0.0
f1 (ppm)

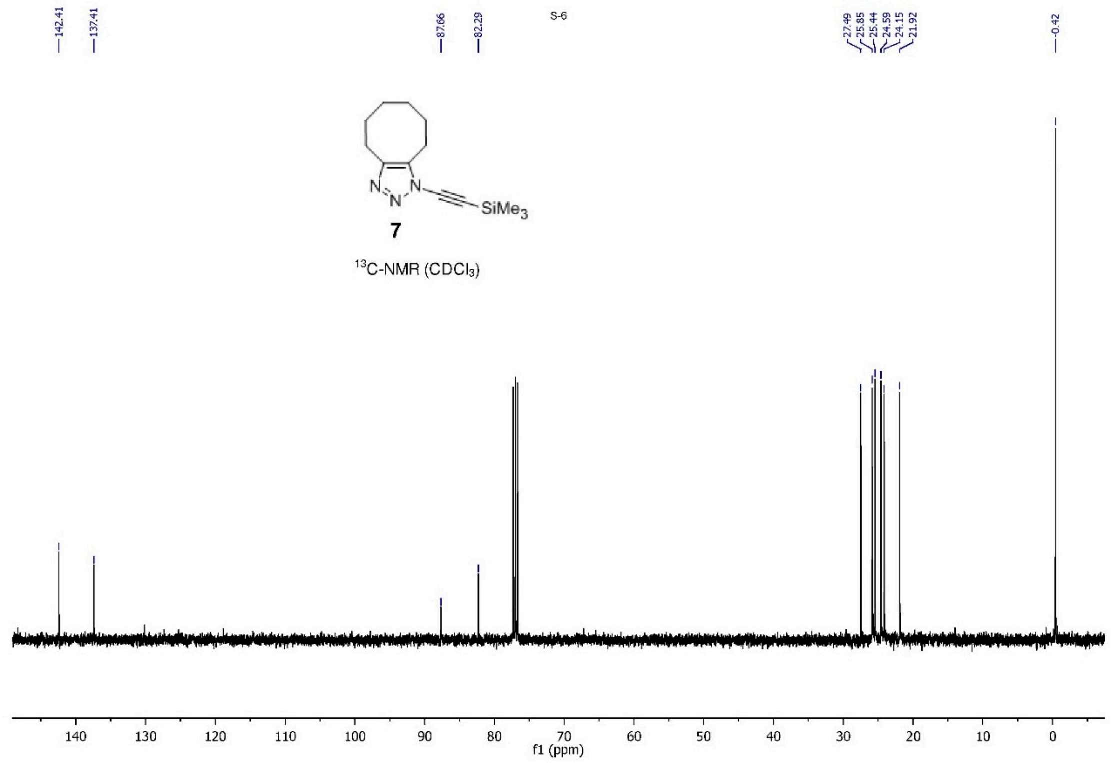
142.41
137.41
87.66
82.29
S-6
27.49
25.85
25.44
24.59
24.15
21.92
-0.42
7
13C-NMR (CDCl3)
140 130 120 110 100 90 80 70 60 50 40 30 20 10 0
f1 (ppm)

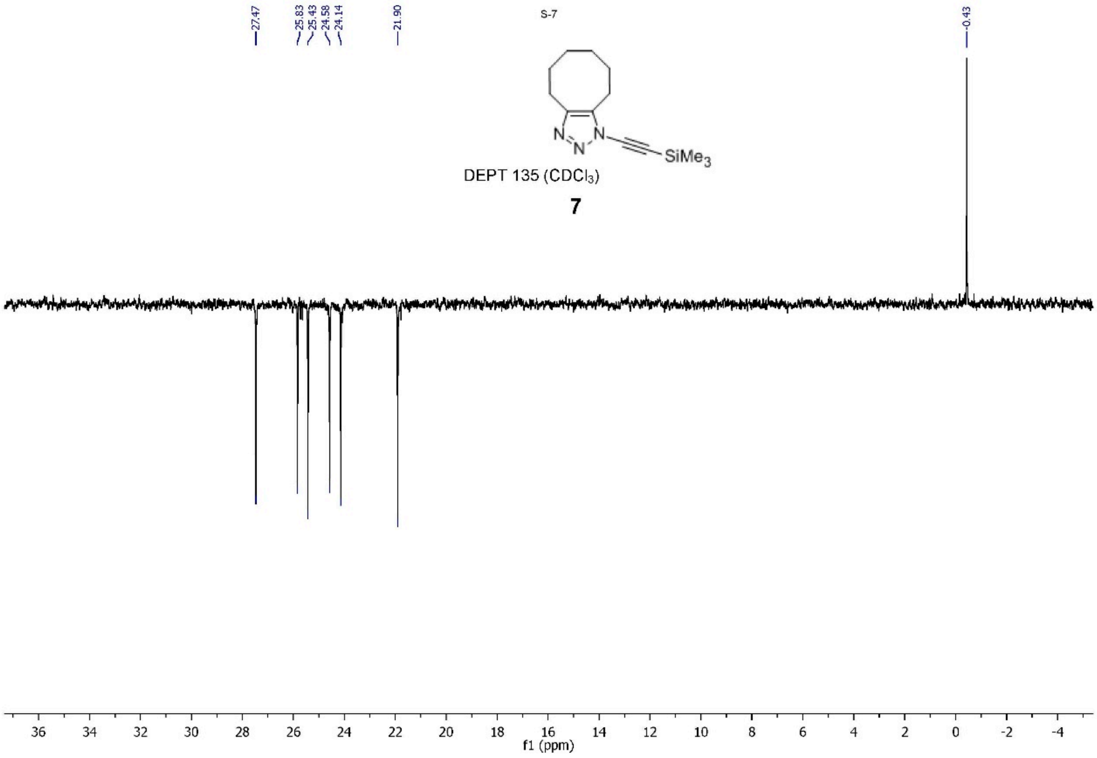

S-7
27.47
25.83
25.43
24.58
24.14
21.90
0.43
N
N
N
SiMe3
DEPT 135 (CDCl3)
7
f1 (ppm)

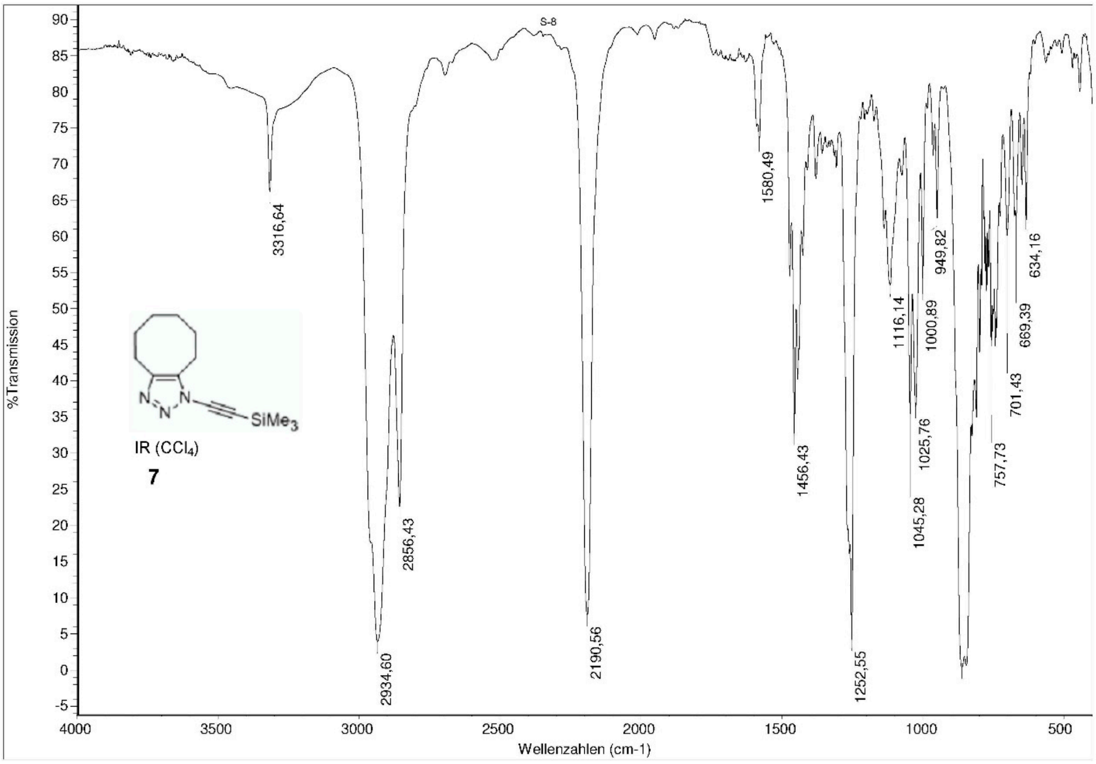

S-8
%Transmission
Wellenzahlen (cm-1)
3316,64
2934,60
2856,43
2190,56
1580,49
1456,43
1252,55
1116,14
1045,28
1025,76
1000,89
949,82
757,73
701,43
669,39
634,16
SiMe3
IR (CCl4)
7

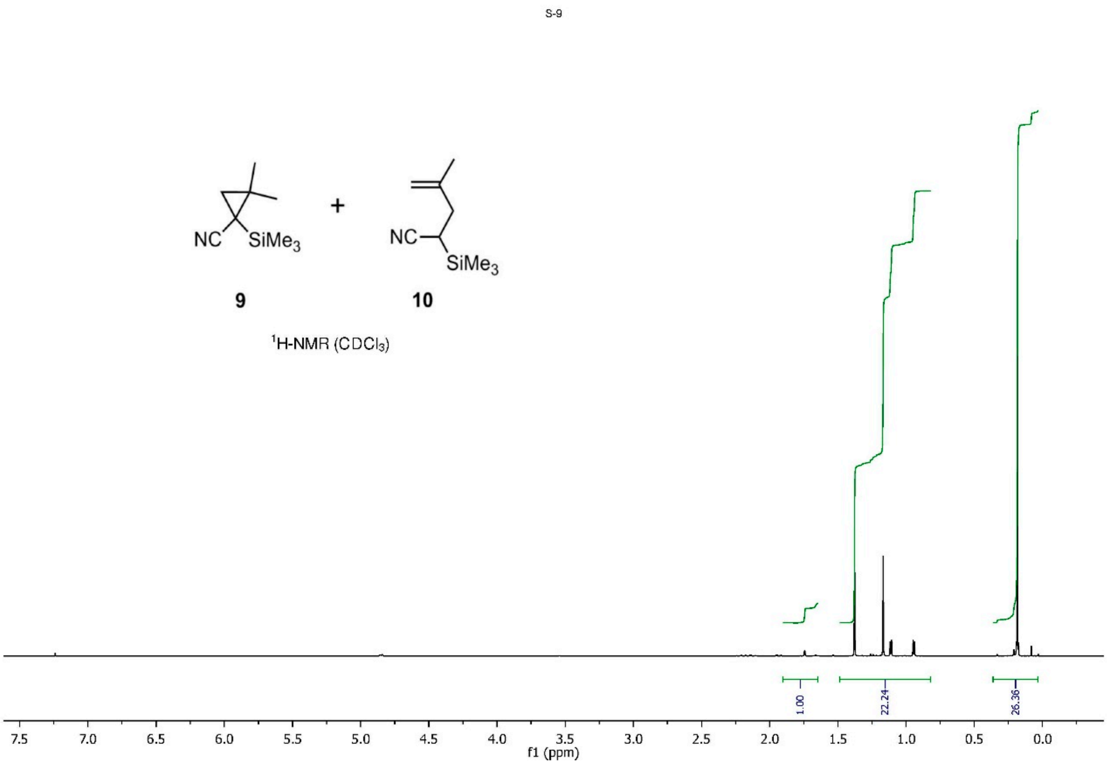
S-9
NC SiMe3
9
NC SiMe3
10
1H-NMR (CDCl3)
1.00
22.24
26.36
f1 (ppm)

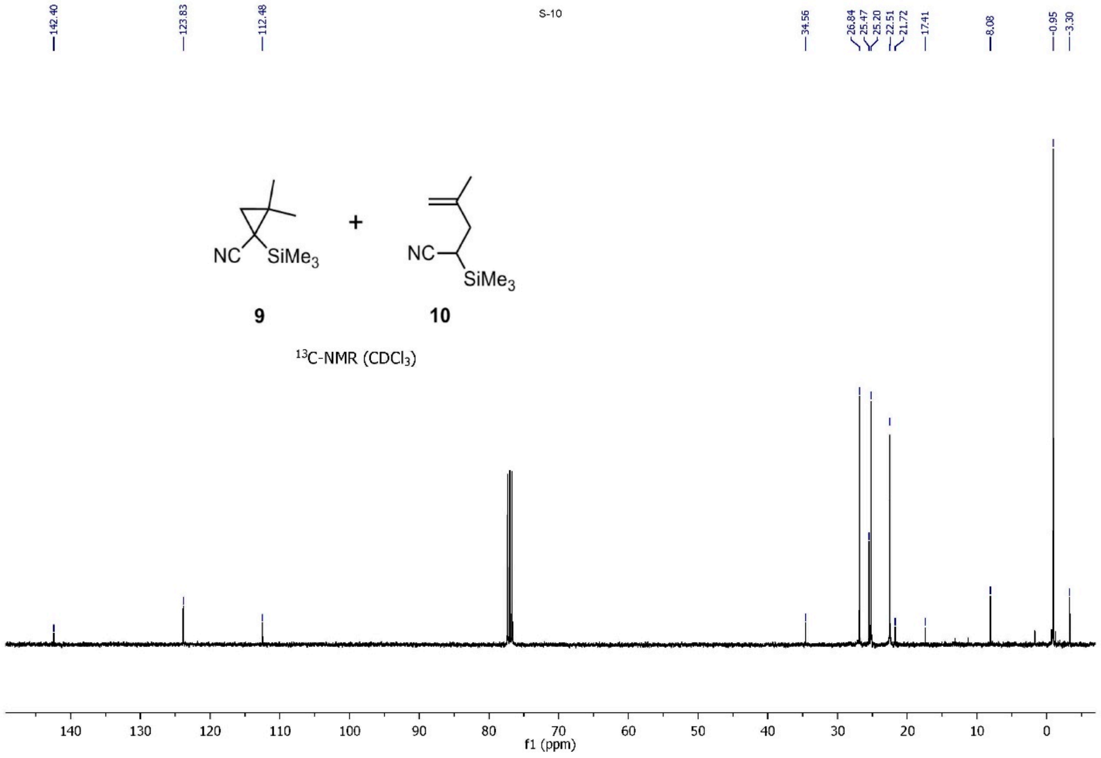
S-10
142.40
123.83
112.48
34.56
26.84
25.47
25.20
22.51
21.72
17.41
8.08
-0.95
-3.30
NC SiMe3
9
NC SiMe3
10
13C-NMR (CDCl3)
f1 (ppm)

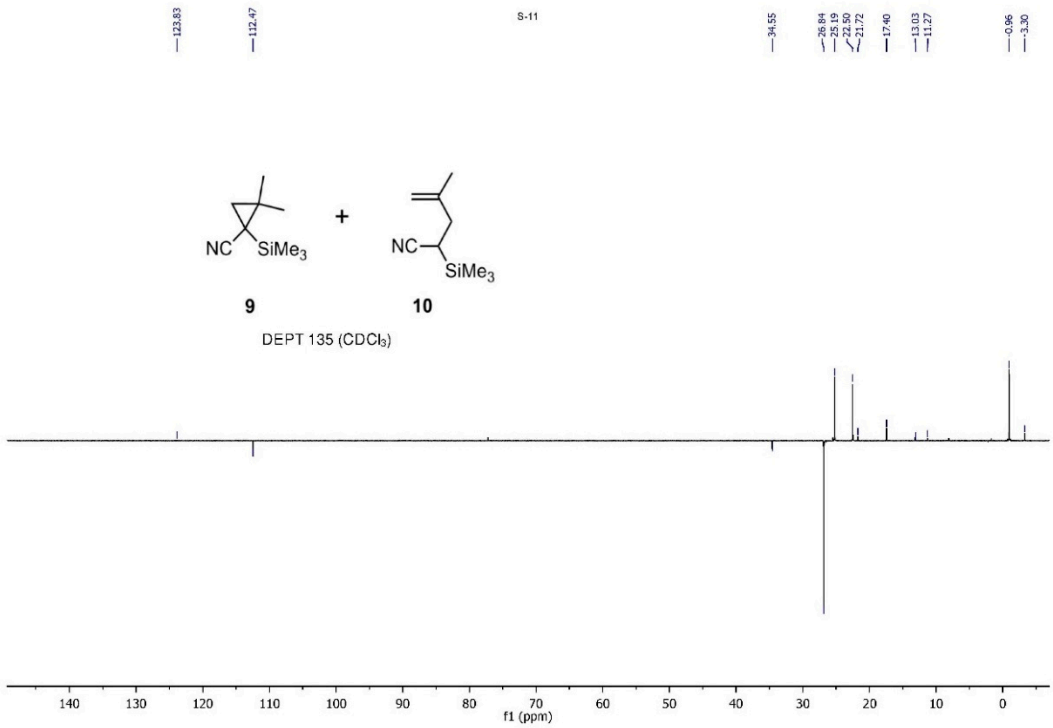

Calculated Atomic Coordinates (in Angstroms) and Energies (in Hatrees) for All Optimized Structures at the B3LYP/6−311++G(3df,3pd) Level of Theory.

**Me3SiCCN3**			
C	−0.00253100	0.03352800	−0.03402300
H	0.02963300	0.03010100	1.05630300
H	1.02498600	0.06430500	−0.39847500
H	−0.49967800	0.95234600	−0.35165100
Si	−0.91049500	−1.47331500	−0.69024000
C	−0.91565800	−1.47814600	−2.56742600
H	−1.42103700	−2.36249000	−2.95746600
H	−1.43311000	−0.59774100	−2.95381800
H	0.10025700	−1.47192900	−2.96414700
C	−2.66685700	−1.51747400	−0.02717000
H	−3.19759900	−2.40358800	−0.37756800
H	−2.67515200	−1.53313400	1.06346100
H	−3.22678600	−0.63893900	−0.35429600
C	−0.02678300	−2.98052900	−0.11712700
C	0.55781500	−3.96699000	0.27337600
N	1.18160000	−5.11703300	0.57095300
N	1.67211600	−5.28950100	1.69543400
N	2.16567600	−5.60399700	2.65550300

Zero-point correction=	0.133726
Thermal correction to Energy=	0.146150
Thermal correction to Enthalpy=	0.147094
Thermal correction to Gibbs Free Energy=	0.093486
Sum of electronic and zero-point Energies=	−649.644515
Sum of electronic and thermal Energies=	−649.632092
Sum of electronic and thermal Enthalpies=	−649.631147
Sum of electronic and thermal Free Energies=	−649.684755

TS: Me_3SiCCN_3 → Me_3SiCCN (^1A) + N_2

C	−0.00019900	0.00073600	−0.00221800
H	0.01427700	−0.00678300	1.08785300
H	1.03104200	0.00902600	−0.35577700
H	−0.47472700	0.92928500	−0.32646700
Si	−0.94916100	−1.47254500	−0.68569900
C	−0.95133100	−1.45114900	−2.56319000
H	−1.44886300	−2.33463700	−2.96576800
H	−1.47795700	−0.57183700	−2.94007600
H	0.06527700	−1.43312100	−2.95719500
C	−2.71518400	−1.48241000	−0.04706000
H	−3.25053200	−2.37097300	−0.38478200
H	−2.73796000	−1.47426400	1.04309400
H	−3.26249900	−0.60793100	−0.40531700
C	−0.06154600	−2.95464700	−0.07793200
C	0.53544100	−3.95702200	0.33706000
N	1.01462900	−5.09261600	0.67079500
N	2.37085400	−4.99683000	1.60638200
N	3.13402100	−5.59449300	2.13539300

Zero-point correction=	0.129460
Thermal correction to Energy=	0.142421
Thermal correction to Enthalpy=	0.143365
Thermal correction to Gibbs Free Energy=	0.088679
Sum of electronic and zero-point Energies=	−649.612836
Sum of electronic and thermal Energies=	−649.599876
Sum of electronic and thermal Enthalpies=	−649.598932
Sum of electronic and thermal Free Energies=	−649.653618

**Me3SiCCN (^{1}A)**			
C	−1.94617500	−0.06463100	−1.37650600
H	−1.73178900	0.66016500	−2.16285800
H	−1.96685000	−1.05538200	−1.82961100
H	−2.93987000	0.15803400	−0.98344500
Si	−0.66738400	0.01961400	−0.00990800
C	−0.93374100	−1.34600200	1.27234800
H	−0.17678300	−1.32283000	2.05511400
H	−1.90822600	−1.17678400	1.73673800
H	−0.93646400	−2.33415900	0.81616600
C	−0.66548000	1.71411900	0.79827900
H	0.08096600	1.77377700	1.59069200
H	−0.44504000	2.49744000	0.07172400
H	−1.64213100	1.93472700	1.23409700
C	0.97051300	−0.52687200	−0.69943200
C	2.20266000	−0.15532000	−0.32626700
N	3.32041500	0.12323700	−0.05720600

Zero-point correction=	0.122023
Thermal correction to Energy=	0.132757
Thermal correction to Enthalpy=	0.133701
Thermal correction to Gibbs Free Energy=	0.086006
Sum of electronic and zero-point Energies=	−540.096939
Sum of electronic and thermal Energies=	−540.086204
Sum of electronic and thermal Enthalpies=	−540.085260
Sum of electronic and thermal Free Energies=	−540.132955

**Me3SiCCN (^{3}A)**			
C	−1.30148100	1.78602500	−0.04420100
H	−0.94497000	2.33889500	0.82522300
H	−0.94291000	2.29604200	−0.93860800
H	−2.39257600	1.82980000	−0.04653600
Si	−0.72800200	−0.00001600	−0.00004400
C	−1.30308700	−0.93110200	−1.52412400
H	−0.94782800	−1.96182800	−1.51735800
H	−2.39422600	−0.95151100	−1.56102700
H	−0.94479100	−0.45675600	−2.43805100
C	−1.30208100	−0.85427200	1.56877500
H	−0.94497500	−1.88340400	1.61370700
H	−0.94457100	−0.33397900	2.45766600
H	−2.39318800	−0.87474600	1.60685900
C	1.11333000	−0.00058300	−0.00029600
C	2.41702200	−0.00024300	−0.00001600
N	3.61426300	−0.00017600	−0.00029800

Zero-point correction=	0.122011
Thermal correction to Energy=	0.132591
Thermal correction to Enthalpy=	0.133535
Thermal correction to Gibbs Free Energy=	0.085232
Sum of electronic and zero-point Energies=	−540.131784
Sum of electronic and thermal Energies=	−540.121204
Sum of electronic and thermal Enthalpies=	−540.120260
Sum of electronic and thermal Free Energies=	−540.168563

**N2**			
N	0.00000000	0.00000000	0.54559400
N	0.00000000	0.00000000	−0.54559400

Zero-point correction=	0.005573
Thermal correction to Energy=	0.007934
Thermal correction to Enthalpy=	0.008878
Thermal correction to Gibbs Free Energy=	−0.012852
Sum of electronic and zero-point Energies=	−109.561799
Sum of electronic and thermal Energies=	−109.559438
Sum of electronic and thermal Enthalpies=	−109.558494
Sum of electronic and thermal Free Energies=	−109.580224

Calculated Atomic Coordinates (in Angstroms) and Energies (in Hatrees) for All Optimized Structures at the CBS-QB3 Level of Theory.

Me_3SiCCN_3

C	−1.89999200	1.91317800	−0.00826300
H	−1.45082600	2.37889900	0.87328000
H	−1.45160000	2.37071800	−0.89447100
H	−2.96781400	2.15431100	−0.00892100
Si	−1.64022100	0.05166000	0.00006200
C	−2.38802000	−0.72526400	−1.53906300
H	−2.21230400	−1.80433100	−1.56134200
H	−3.46977300	−0.55970100	−1.57014400
H	−1.95545900	−0.29720600	−2.44752700
C	−2.38645200	−0.71145700	1.54682800
H	−2.21229600	−1.79056800	1.57764200
H	−1.95166700	−0.27663100	2.45100200
H	−3.46790400	−0.54401700	1.57842700
C	0.16998100	−0.27455000	0.00034100
C	1.36850200	−0.47061700	0.00043700
N	2.66342900	−0.82642400	−0.00031900
N	3.55445100	0.04011800	0.00006400
N	4.48477900	0.68024100	0.00018500

CBS-QB3 (0 K)=	−648.560873
CBS-QB3 Energy=	−648.548275
CBS-QB3 Enthalpy=	−648.547331
CBS-QB3 Free Energy=	−648.601778

TS: $Me_3SiCCN_3 \rightarrow Me_3SiCCN$ (^1A) + N_2

C	0.00000000	0.00000000	0.00000000
H	0.00000000	0.00000000	1.09295600
H	1.03829400	0.00000000	−0.34131200
H	−0.46605100	0.93136200	−0.33772400
Si	−0.95043400	−1.47461300	−0.68374700
C	−0.95088800	−1.45364000	−2.56302600
H	−1.45164000	−2.33808300	−2.96708700
H	−1.47577000	−0.57066300	−2.94217900
H	0.06882100	−1.43892900	−2.95681900
C	−2.71840800	−1.48202800	−0.04501700
H	−3.25693900	−2.37175900	−0.38357800
H	−2.74094600	−1.47413200	1.04787100
H	−3.26571800	−0.60427200	−0.40407500
C	−0.06484500	−2.95645000	−0.07071300
C	0.53340800	−3.96098900	0.34686300
N	1.01779800	−5.09361500	0.69182500
N	2.38878200	−4.99226100	1.59722600
N	3.15398200	−5.60519300	2.11672300

CBS-QB3 (0 K)=	−648.525116
CBS-QB3 Energy=	−648.512065
CBS-QB3 Enthalpy=	−648.511121
CBS-QB3 Free Energy=	−648.566105

Me3SiCCN (^{1}A)			
C	−1.95298800	−0.07073800	−1.37068700
H	−1.74272500	0.64997900	−2.16591300
H	−1.97811200	−1.06711700	−1.81789600
H	−2.94659900	0.15681900	−0.97193800
Si	−0.66353700	0.02009200	−0.01158400
C	−0.93774800	−1.32891200	1.28954200
H	−0.17818900	−1.29931600	2.07368500
H	−1.91344200	−1.14929200	1.75418100
H	−0.94553100	−2.32556100	0.84523400
C	−0.64802300	1.72370600	0.78180800
H	0.11017300	1.78798800	1.56670300
H	−0.43387700	2.50322700	0.04499600
H	−1.62134000	1.95098000	1.22898300
C	0.96498200	−0.55082400	−0.70434500
C	2.19687300	−0.16467300	−0.32841200
N	3.31436900	0.12280600	−0.05761300

CBS-QB3 (0 K)=	−539.178361
CBS-QB3 Energy=	−539.167577
CBS-QB3 Enthalpy=	−539.166633
CBS-QB3 Free Energy=	−539.214460

Me3SiCCN (^{3}A)			
C	1.30330700	−1.10501300	−1.40602700
H	0.94479400	−2.12965800	−1.27811200
H	0.94419500	−0.73929900	−2.37148100
H	2.39724700	−1.13212900	−1.44111300
Si	0.72858400	0.00006600	0.00000900
C	1.30547400	1.76964500	−0.25319500
H	0.94833900	2.42254500	0.54738200
H	2.39949200	1.81185400	−0.26034600
H	0.94651500	2.17281000	−1.20367500
C	1.30243600	−0.66610300	1.65980300
H	0.94377000	−0.04372500	2.48370800
H	0.94326500	−1.68517600	1.82489100
H	2.39632900	−0.68301600	1.70114000
C	−1.11264400	0.00134200	−0.00047800
C	−2.41986500	0.00054300	−0.00039500
N	−3.61948000	0.00034100	−0.00011100

CBS-QB3 (0 K)=	−539.211421
CBS-QB3 Energy=	−539.200781
CBS-QB3 Enthalpy=	−539.199837
CBS-QB3 Free Energy=	−539.248286

N2			
N	0.00000000	0.00000000	0.54772100
N	0.00000000	0.00000000	−0.54772100

CBS-QB3 (0 K)=	−109.398445
CBS-QB3 Energy=	−109.396085
CBS-QB3 Enthalpy=	−109.395141
CBS-QB3 Free Energy=	−109.416878
